
JOURNAL INFORMATION
==============================
NLM Title Abbreviation: Daniels Tex Med J
NLM Journal ID: 0006376

Daniel's Texas Medical Journal

ISSN: 0892-8487
Publisher: Biomedical Journal Digitization Project

ARTICLE INFORMATION
==============================
PMCID: PMC9162594
Article version: 1
Subjects: Bibliography

The Best Antiseptic, Listerine

Print publication date: 1887 Feb
Volume: 2
Issue: 8
First page: 352
Last page: 352
License: This work is free of known copyright restrictions. For more information, please see PMC Back Issue Digitization.
License URL: https://creativecommons.org/publicdomain/mark/1.0/

==============================

DISEASES OF THE BLOOD AND NUTRITION, AND INFECTIOUS DISEASES; being Vol. IV. of "A Handbook of Practical Medicine," by Dr. Hermann Eichhorst, and Vol. XII. of Wood's Library for 1886 (completing the set, price of set, $15.00). Illustrated. New York, William Wood & Company.

These two works complete the series of the "Library" for 1886. We have not given them a critical examination, but a casual glance reveals the fact that in making the selection the publishers exercised commendable discretion and taste in their endeavor to give great value for little money, to give variety, and at the same time to have all of the works of a high order of excellence.

---

THE BEST ANTISEPTIC, for both internal and external use. Listerine; antiseptic, prophylactic, deodorant, non-toxic, non-irritant, non-escharotic. Absolutely safe. Agreeable, scientific, and strictly professional. Lambert Pharmacal Co., St Louis, Missouri, 1887.

Such is the title of a most RECHERCHE little quarto of 32 pages, gotten up by Mr. Lambert, who is so generally known as the "*Doctor's friend.*" We do not know that we can say of it, after a careful reading, anything more to the point, or more deserved, or say it in any better language than that used by Mr. L. in a letter to us. "It is an aggregation of authorities that certainly leaves no room to question the scientific value of Listerine, and defines its true pharmaceutical field from every standpoint. The microscopic tables clearly prove our consistency in presenting Listerine as a true and safe antiseptic specially for internal and external use about the human system."

Our readers will enjoy a treat in reading this little keepsake, which they can get by dropping a postal card to Mr. Lambert, 116 Olive St., St. Louis, Mo.

---

### ADVERTISERS' NOTICES.

"NESTLE'S FOOD" is no longer on probation. It is prescribed in all parts of the world by those physicians who are progressive and who appreciate the scientific principles upon which the food is constructed, and competent authority pronounce it a perfect substitute for mothers milk.
